# En Route to Personalised Medicine in Obstructive Sleep Apnoea

**DOI:** 10.3390/jcm12103457

**Published:** 2023-05-14

**Authors:** Andras Bikov

**Affiliations:** 1 North West Lung Centre, Wythenshawe Hospital, Manchester University NHS Foundation Trust, Manchester M23 9LT, UK; andras.bikov@gmail.com; 2 Division of Infection, Immunity and Respiratory Medicine, University of Manchester, Manchester M13 9MT, UK

Obstructive sleep apnoea (OSA) is a common disorder that can cause night- and daytime symptoms and impair driving and work performance. It is also a risk factor for cardiovascular, metabolic, and mental health diseases. Continuous positive airway pressure (CPAP) is the first-line treatment for OSA; however, it has failed to prove its efficacy in reducing cardiovascular morbidity and mortality in randomised controlled trials (RCTs) [1,2]. This Special Issue focuses on three potential reasons for this failure. These include (1) the incomplete understanding of the molecular links between OSA and comorbidities, (2) the lack of biomarkers for predicting which patient is at high risk for developing comorbidities, and (3) the incomplete understanding of the factors associated with adherence to treatment.

OSA is frequently related to obesity, which can independently lead to cardiovascular disease (CVD). On the one hand, weight loss is associated with a lower risk for OSA and a reduction in drowsiness [3]. On the other hand, weight loss, in parallel with CPAP, was reported to improve cardiometabolic outcomes in patients with OSA [4]. Weight changes are strongly related to appetite. Pardak et al. investigated the overnight variation of hormones regulating appetite in OSA patients. The authors found significantly lower ghrelin values in OSA patients, but only at 5 and 7 am. The ghrelin levels were primarily determined by the patients’ body mass indices rather than hypoxaemia [5]. Several mechanisms lead to dyslipidaemia in OSA patients, and dyslipidaemia directly prompts CVD [6]. Meszaros et al. have reported reduced levels of soluble low-density lipoprotein receptor-related protein-1 in patients with OSA, suggesting that the impaired disposal of atherogenic lipoproteins potentially contributes to high CVD risk [7]. Comorbid insomnia in sleep apnoea (COMISA) poses an additional risk factor for cardiovascular diseases and can limit adherence to CPAP [8]. However, the pathophysiology of COMISA has not been fully explored at the molecular level. Gabryelska investigated brain-derived neurotrophic factor (BDNF) and found that this molecule is associated with COMISA [9]. Further studies are needed to explore the role of this promising biomarker. Most importantly, the link between OSA and CVD is not direct and may be influenced by comorbid conditions. These conditions, such as chronic obstructive pulmonary disease (COPD), are associated with inflammation. Chaszczewska-Markowska et al. investigated numerous cytokines and their associations with the comorbidities of OSA patients. Their mapping exercise may help us to understand the role of specific inflammatory molecules in OSA and highlight the importance of controlling comorbid conditions [10].

Rapid-eye movement (REM)-predominant OSA is associated with higher cardiovascular risk [11]. As REM phases are more likely to occur in the later stages of the sleep, suboptimal CPAP usage does not cover this period. Therefore, screening for REM-related OSA holds important clinical value. Balcan et al. investigated the characteristics of REM-related OSA and reported that it is associated with a high BMI and the female sex [12]. Atherogenic index of plasma (AIP) is a promising biomarker to predict cardiovascular disease in a general population. We found that although AIP was different in OSA patients compared to controls and was related to disease severity, it offered limited additional value with respect to predicting cardiovascular disease, hypertension, and diabetes compared to high-density lipoprotein cholesterol [13]. Plasma cysteine levels were also reported to relate to cardiovascular disease in the general population. Although the differences in cysteine levels were insignificant when comparing patients with OSA and healthy controls, they were correlated with markers of sleep-related hypoxaemia and, most importantly, blood pressure values. In addition, the use of CPAP resulted in significant reductions in plasma cysteine concentrations [14]. The study highlights two important aspects. First, perhaps markers associated with CVD, but not directly linked to OSA, should guide our treatment when mitigating the risk of CVD. Second, we should focus on hypoxaemia rather than the apnoea–hypopnoea index (AHI) when estimating the likelihood of cardiovascular disease for OSA patients. This recommendation is well supported by the study by Gabryelska et al., who reported a strong relationship between sleep-related hypoxaemia and type 2 diabetes mellitus [15]. Patients with chronic heart disease can develop both obstructive and central sleep apnoea. In their prospective study, Naito et al. reported that the severity of central apnoea is independently related to significant cardiovascular events [16]. This emphasises that distinguishing central and obstructive events determining AHI could yield important clinical information. Most studies on OSA have focused on coronary artery disease, atrial fibrillation, and subsequent left heart failure [17]. As chronic hypoxaemia may lead to the development of pulmonary hypertension [18], it is plausible that patients with biventricular failure are at a higher risk for cardiovascular complications. The comprehensive review by Tadic and Cuspidi focused on the echocardiographic markers of right heart function and their abnormalities in OSA [19].

The adherence to CPAP treatment in the largest RCTs was suboptimal [1,2]. In a real-life population in the United Kingdom, adherence to CPAP was most strongly associated with driving status, and it did not relate to disease severity or daytime sleepiness [20]. This could prompt a patient-centred approach to improving adherence. Telemedicine could be a potential tool in this regard, as shown by O’Connor-Reina et al [21]. However, it is recognised that some patients are intolerant to CPAP despite multiple interventions. Cephalometrics can be used for tailoring second-line treatment, such as oral appliances, myofunctional therapy, or upper airway surgery [22].

In summary, the reason behind the negative RCTs include a lack of understanding of the link between OSA and CVD, a lack of clinically useful biomarkers, and suboptimal adherence. I believe this Special Issue has helped, at least partly, to reveal these uncertainties. These articles also highlight that OSA should not be treated as a blanket disease and that, as clinicians, we can use molecular and physiological phenotypes to improve patient outcomes.
